# Corneal Parameters in Healthy Subjects Assessed by Corvis ST

**DOI:** 10.18502/jovr.v15i1.5936

**Published:** 2020-02-02

**Authors:** Ramin Salouti, Mansoureh Bagheri, Anis Shamsi, Mohammad Zamani

**Affiliations:** ^1^Poostchi Ophthalmology Research Center, Department of Ophthalmology, School of Medicine, Shiraz University of Medical Sciences, Shiraz, Iran; ^2^Salouti Cornea Research Center, Salouti Eye Clinic, Shiraz, Iran; ^3^Health Policy Research Center, Shiraz University of Medical Sciences, Shiraz, Iran

**Keywords:** Central Corneal Thickness, Corneal Biomechanics, Corvis ST, Intraocular Pressure

## Abstract

**Purpose:**

To evaluate corneal biomechanics using Corvis ST in healthy eyes from Iranian keratorefractive surgery candidates.

**Methods:**

In this prospective consecutive observational case series, the intraocular pressure (IOP), central corneal thickness (CCT), and biomechanical properties of 1,304 eyes from 652 patients were evaluated using Corvis ST. Keratometric readings and manifest refraction were also recorded.

**Results:**

The mean (±SD) age of participants was 28 ± 5 years, and 31.7% were male. The mean spherical equivalent refraction was –3.50 ± 1.57 diopters (D), the mean IOP was 16.8 ± 2.9 mmHg, and the mean CCT was 531 ± 31 μm for the right eye. The respective means (±SD) corneal biomechanical parameters of the right eye were as follows: first applanation time: 7.36 ± 0.39 milliseconds (ms); first applanation length: 1.82 ± 0.22 mm; velocity in: 0.12 ± 0.04 m/s; second applanation time: 20.13 ± 0.48 ms; second applanation length: 1.34 ± 0.55 mm; velocity out: –0.67 ± 0.17 m/s; total time: 16.84 ± 0.64 ms; deformation amplitude: 1.05 ± 0.10 mm; peak distance: 4.60 ± 1.01 mm; and concave radius of curvature: 7.35 ± 1.39 mm. In the linear regression analysis, IOP exhibited a statistically significant association with the first and second applanation times, total time, velocity in, peak distance, deformation amplitude, and concave radius of curvature.

**Conclusion:**

Our study results can be used as a reference for the interpretation of Corvis ST parameters in healthy refractive surgery candidates in the Iranian population. Our results confirmed that IOP is a major determinant of Corvis parameters.

##  INTRODUCTION

The cornea has a complex biomechanical structure that determines its response under stress conditions.^[1]^ Currently, ophthalmologists are deeply interested in characterizing the corneal biomechanical properties in pathological conditions and after refractive surgery.^[2]^ Furthermore, corneal biomechanics affect intraocular pressure (IOP) measurement and may also be an important risk factor for the development of glaucomatous optic neuropathy.^[3]^

To date, only two devices have been designed to evaluate corneal biomechanical properties *in vivo:* the Ocular Response Analyzer (ORA; Reichert Ophthalmics, Depew, NY), a dynamic bidirectional applanation device and the Corvis ST (Oculus Optikgeräte GmbH, Wetzlar, Germany), a dynamic non-contact Scheimpflug analyser device.^[2]^ Both devices use an air pulse to impress the cornea.^[4]^ In contrast to the ORA, which cannot display the dynamics of the corneal deformation process in real time, the Corvis ST uses the real-time corneal deformation data to analyze corneal biomechanics. To accomplish this, Corvis ST captures a series of horizontal Scheimpflug images using a high-speed camera that gathers 4,300 frames per sec within a 100 milliseconds (ms) period.^[1,5]^

Currently, there are few reports regarding the normal distribution of Corvis ST parameters from different populations.[1, 6–10] Because ethnicity is a known determinant of corneal biomechanical properties,^[11]^ the normative database from various populations are very useful and can guide us in spotting abnormal cases. The aim of this study was to evaluate the corneal biomechanical properties using the Corvis ST in healthy eyes from Iranian patients who have been evaluated for keratorefractive surgery.

##  METHODS

##  Study Population

In this prospective case series, which was conducted from January 2012 to December 2013, corneal biomechanical parameters from Corvis ST were recorded for 1,304 eyes from 652 consecutive healthy keratorefractive surgery candidates with no eye disorders except myopia. A complete eye examination, including visual acuity measurement, slit-lamp biomicroscopy, and fundus exam using a 90-diopter noncontact lens was performed on each eye. Cases with positive history (or objective signs) of ocular disorders (e.g., glaucoma, uveitis, corneal ectatic disorders, Fuchs's corneal dystrophy, and diabetic retinopathy), chronic use of topical medications, previous ocular surgery, corneal scars or opacities, irregular astigmatism, systemic diseases, or inability to cooperate with any measurement device were excluded. The research protocol adhered to the tenets of the Declaration of Helsinki and detailed informed consent was signed by all individuals. The study protocol was approved by the Ethics Committee at the Shiraz University of Medical Sciences.

##  Measurements

Refraction was measured using an autorefractometer (Canon R-50; Canon Inc., Tokyo, Japan), and keratometric measurements were recorded from Pentacam HR (Oculus Optikgeräte GmbH, Wetzlar, Germany) scan reports.

Ocular biomechanical parameters, IOP, and central corneal thickness (CCT) were obtained using Corvis ST. Corvis ST measures the biomechanical response of the cornea at the moment of the first and second applanations, and highest concavity events. IOP is calculated based on the timing of the first applanation event.^[11]^ Corvis ST measures and records the time to reach applanation (T1, T2), the length of the flattened segment in a Scheimpflug image (L1, L2), and corneal movement velocity during applanation (V1, V2) at the moment of both first and second applanations, respectively. It also measures the total time (T), deformation amplitude (DA), distance between bending points of the cornea (PD), and the concave radius of curvature (R) at the point of highest concavity. All of the described Corvis ST parameters were recorded for analysis.

Each instrument was calibrated at the outset of the study, and then at regular intervals (as per manufacturer recommendations). All measurements from each device were performed by the same qualified operator using the criteria provided by the devices manufacturer.

##  Statistical Analysis

Data were analyzed using IBM SPSS Statistics software version 21 (SPSS Inc., Chicago, IL) and MedCalc version 12.2.1 (*MedCalc* Software, Mariakerke, Belgium). Descriptive statistical results were reported as mean ± standard deviation (SD). Corvis ST data were presented as mean and normal range (mean ± 1.96 SD).

Only data from the right eyes of participants were used for regression analysis. Factors with *P* < 0.05 in simple linear regression analysis were entered into a multiple stepwise linear regression analysis. *P* < 0.05 was considered to be statistically significant.

##  RESULTS

The mean (± SD) age of patients was 28 ± 5 years (range: 20 to 47 years), and 31.7% were male. The majority of the enrolled cases were Persian. The baseline characteristics of both eyes are presented in Table 1. The mean and normal range of Corvis ST parameters along with the evaluation of absolute and relative variations between right and left eyes are shown in Table 2.

**Table 1:** Baseline characteristics of the study cohort


	**Right eyes${}^{a}$**	**Left eyes${}^{a}$**
SE, D	–3.50 ± 1.57	–3.47 ± 1.59
Km, D	43.7 ± 1.3	43.7 ± 1.3
Ka, D	1.2 ± 0.8	1.2 ± 0.8
CCT, μm	531 ± 31	531 ± 31
IOP, mmHg	16.8 ± 2.9	16.6 ± 2.7
	
	
${}^{a}$Data are presented as the mean ± standard deviation.	
CCT, central corneal thickness; D, diopter; IOP, intraocular pressure; Ka, astigmatic keratometry; Km, mean keratometry; SE, spherical equivalent refraction		

**Table 2:** Mean and normal range of Corvis parameters among participants


	**Right eyes${}^{a}$**	**Left eyes${}^{a}$**	**Absolute variation${}^{b}$ (95% range)**	**Relative variation${}^{c}$ (95% range)**
T1, milliseconds	7.36 (6.60 to 8.12)	7.34 (6.61 to 8.07)	± 0.66	± 9.0%
L1, mm	1.82 (1.37 to 2.23)	1.83 (1.38 to 2.28)	± 0.095	± 5.2%
V1, m/s	0.12 (0.04 to 0.20)	0.12 (0.04 to 0.20)	± 0.65	± 565%
T2, milliseconds	20.13 (19.19 to 21.07)	20.15 (19.23 to 21.07)	± 0.58	± 2.9%
L2, mm	1.34 (0.26 to 2.42)	1.38 (0.26 to 2.50)	± 0.40	± 29.4%
V2, m/s	–0.67 (–1.00 to –0.34)	–0.67 (–1.06 to –0.28)	± 1.45	± 216%
T, milliseconds	16.84 (15.59 to 18.09)	16.88 (15.70 to 18.06)	± 1.29	± 7.7%
DA, mm	1.05 (0.85 to 1.25)	1.06 (0.86 to 1.26)	± 0.19	± 12.3%
PD, mm	4.60 (2.62 to 6.58)	4.63 (2.66 to 6.58)	± 2.56	± 55.5%
R, mm	7.35 (4.63 to 10.07)	7.35 (4.34 to 10.37)	± 3.29	± 44.8%
${}^{a}$Data are presented as the mean (95% range)				
${}^{b}$Calculated as: ± 1.96 SD of the mean difference (right–left)				
${}^{c}$Calculated as: ± [(1.96 SD of the mean difference)/(mean value of both eyes)] * 100				
DA, deformation amplitude; L1, length of applanation 1; L2, length of applanation 2; PD, peak distance; R, radius; T, time of highest concavity; T1, time of applanation 1; T2, time of applanation 2; V1, velocity of applanation 1; V2, velocity of applanation 2

**Table 3:** Linear regression analysis demonstrating association between selected demographic and ocular factors to each Corvis ST parameters at the first applanation moment


	**T1**		**L1**		**V1**	
	**SC**	**** ***P*** **-value${}^{a}$**	**SC**	**** ***P*** **-value${}^{a}$**	**SC**	**** ***P*** **-value${}^{a}$**
Age			
Sex (male to female)			–0.091	0.021${}^{b}$
SE			
Km			
Ka			
CCT	0.403	< 0.001${}^{b}$		
IOP	0.964	< 0.001${}^{b}$		–0.328	< 0.001${}^{b}$
	
	
${}^{a}$Only factors with *P*-value < 0.05 in simple linear regression analysis are shown here	
${}^{b}$Denotes factors that remained significant after multiple stepwise linear regression analysis						
CCT, central corneal thickness; IOP, intraocular pressure; Ka, astigmatic keratometry; Km, mean keratometry; L1, length of applanation 1; SC, standardized coefficient; SE, spherical equivalent refraction; T1, time of applanation 1; V1, velocity of applanation 1						

**Table 4:** Linear regression analysis demonstrating association between selected demographic and ocular factors to each Corvis ST parameters at the second applanation moment


	**T2**		**L2**		**V2**	
	**SC**	**** ***P*** **-value${}^{a}$**	**SC**	**** ***P*** **-value${}^{a}$**	**SC**	**** ***P*** **-value${}^{a}$**
Age			
Sex (male to female)			
SE			
Km		–0.092	0.021${}^{b}$	
Ka		–0.117	0.003${}^{b}$	
CCT		0.134	0.001${}^{b}$	0.107	0.007${}^{b}$
IOP	–0.568	< 0.001${}^{b}$		
	
	
${}^{a}$Only factors with *P*-value < 0.05 in simple linear regression analysis are shown here						
${}^{b}$Denotes factors that remained significant after multiple stepwise linear regression analysis						
CCT, central corneal thickness; IOP, intraocular pressure; Ka, astigmatic keratometry; Km, mean keratometry; L2, length of applanation 2; SC, standardized coefficient; SE, spherical equivalent refraction; T2, time of applanation 2; V2, velocity of applanation 2						

**Table 5:** Linear regression analysis demonstrating an association between selected demographic and ocular factors to each Corvis ST parameters at the highest concavity moment


	**T**		**DA**		**PD**		**R**	
	**SC**	**** ***P*** **-value${}^{a}$**	**SC**	**** ***P*** **-value${}^{a}$**	**SC**	**** ***P*** **-value${}^{a}$**	**SC**	**** ***P*** **-value${}^{a}$**
Age				
Sex (male to female)	0.125	0.001${}^{b}$			
SE	0.086	0.028${}^{b}$			
Km				
Ka				
CCT		–0.218	< 0.001		0.211	< 0.001${}^{b}$
IOP	0.135	0.001${}^{b}$	–0.651	< 0.001${}^{b}$	–0.241	< 0.001${}^{b}$	0.189	< 0.001${}^{b}$
${}^{a}$Only factors with *P* < 0.05 in simple linear regression analysis are shown here								
${}^{b}$Denotes factors that remained significant after multiple stepwise linear regression analysis								
CCT, central corneal thickness; DA, deformation amplitude; IOP, intraocular pressure; Ka, astigmatic keratometry; Km, mean keratometry; PD, peak distance; R, radius; SC, standardized coefficient; SE, spherical equivalent refraction; T, time of highest concavity								

**Table 6:** Mean and normal range of Corvis parameters categorized based on the intraocular pressure


	**Intraocular Pressure, mm Hg${}^{a}$**			
	**10.00–12.99 (** ***n*** ** = 30)**	**13.00–15.99 (** ***n*** ** = 230)**	**16.00–18.99 (** ***n*** ** = 252)**	**19.00–22.00 (** ***n*** ** = 104)**
T1, milliseconds${}^{b}$	6.78 (6.54 to 7.01)	7.06 (6.76 to 7.37)	7.39 (7.10 to 7.68)	7.80 (7.46 to 8.14)
V1, m/s${}^{b}$	0.114 (0.042 to 0.186)	0.130 (0.074 to 0.185)	0.117 (0.050 to 0.184)	0.080 (0.020 to 0.140)
T2, milliseconds${}^{b}$	21.21 (20.75 to 21.68)	20.32 (19.43 to 21.20)	20.01 (19.34 to 20.69)	19.87 (19.27 to 20.48)
T, milliseconds${}^{b}$	16.16 (15.47 to 16.86)	16.80 (15.48 to 18.11)	16.93 (15.74 to 18.12)	16.86 (15.82 to 17.91)
DA, mm${}^{b}$	1.15 (1.04 to 1.26)	1.11 (0.942 to 1.28)	1.04 (0.908 to 1.17)	0.989 (0.764 to 1.21)
PD, mm${}^{b}$	5.46 (5.11 to 5.81)	4.77 (2.81 to 6.73)	4.49 (2.50 to 6.49)	4.36 (2.41 to 6.30)
R, mm${}^{b}$	7.42 (3.53 to 11.31)	7.13(4.29 to 9.97)	7.35 (4.75 to 9.95)	7.47 (6.09 to 8.86)
${}^{a}$Data are presented as the mean (95% range); only analyses of right eyes are shown here				
${}^{b}$Only parameters that have shown significant association with IOP are presented here				
DA, deformation amplitude; PD, peak distance; R, radius; T, time of highest concavity; T1, time of applanation 1; T2, time of applanation 2; V1, velocity of applanation 1				

**Figure 1:**
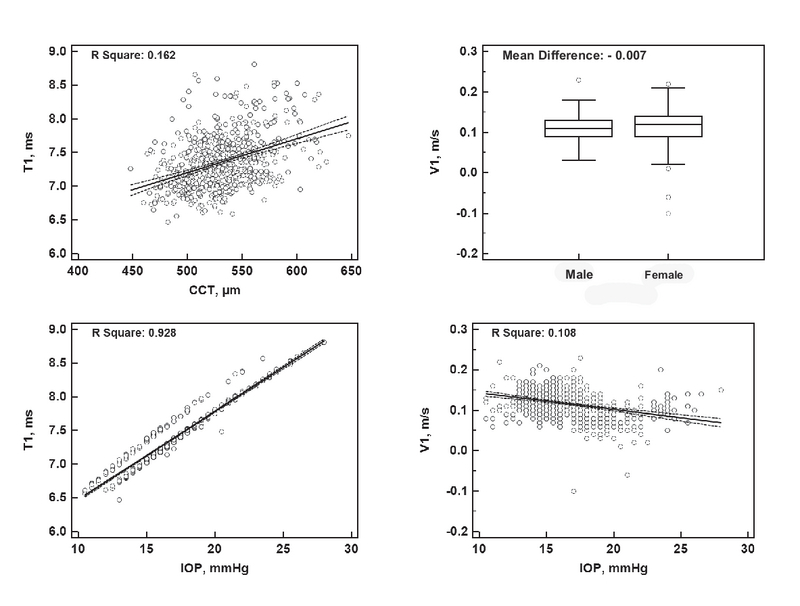
Significant determinants of the selected Corvis ST parameters at the first applanation moment. CCT, central corneal thickness; IOP, intraocular pressure.

**Figure 2:**
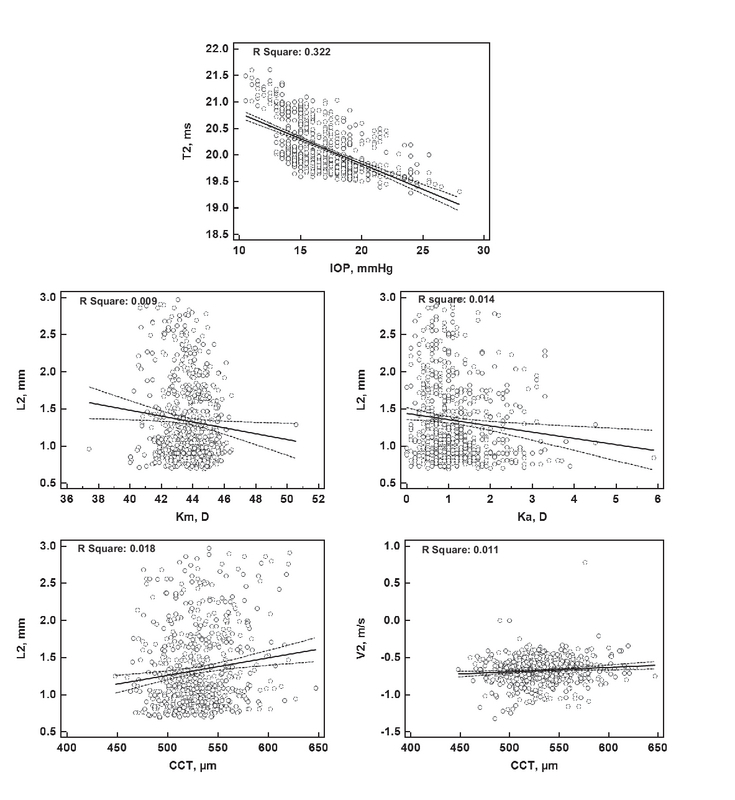
Significant determinants of the selected Corvis ST parameters at the second applanation moment. CCT, central corneal thickness; IOP, intraocular pressure; Km, mean keratometry; Ka, astigmatic keratometry.

**Figure 3:**
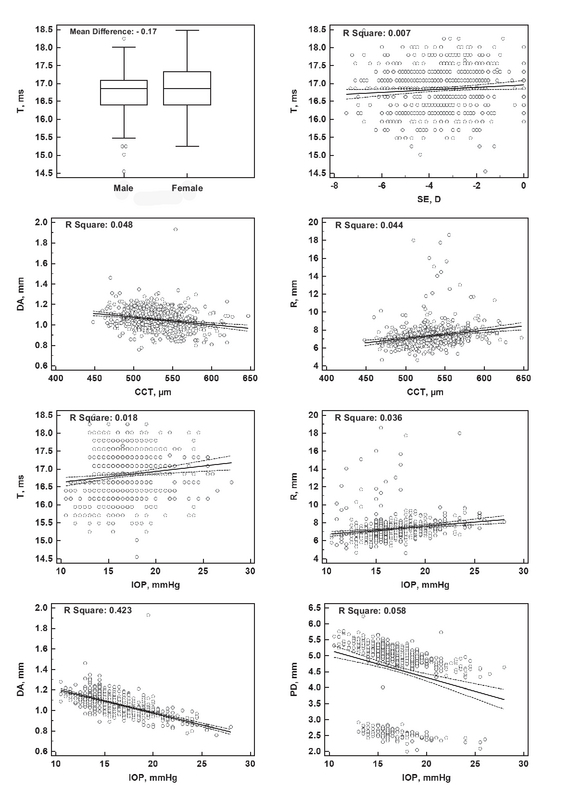
Significant determinants of the selected Corvis ST parameters at the highest concavity moment. CCT, central corneal thickness; IOP, intraocular pressure; SE, spherical equivalent refraction.

Linear regression analysis was used to evaluate the possible association between different demographic and ocular factors with each Corvis ST parameter [Tables 3–5, and Figures 1–3]. The most important and clinically relevant associations [with Standardized Coefficient (SC) > 0.3] were as follows: IOP (SC: 0.964) and CCT (SC: 0.403) for T1; IOP (SC: –0.328) for V1; IOP (SC: –0.568) for T2; and IOP (SC: –0.651) for DA. Table 6 presents the normal values of Corvis ST parameters, stratified based on the corresponding IOP.

Considering gender, only V1 and T have independently been influenced by the gender. The mean V1 and T was 0.110 ± 0.033 vs 0.117 ± 0.037 m/s (*P* = 0.021) and 16.7 ± 0.6 vs 16.9 ± 0.6 ms (*P* = 0.001) for the males vs females, respectively.

##  DISCUSSION

Corvis ST biomechanical parameters are some geometrical factors that are generated during inward and then outward movements of the cornea after a single puff of air and are essentially determined as a product of three different factors: the air puff pressure, the IOP, and the corneal biomechanical properties. Air puff pressure is constant in all cases, and the incident apparent IOP could be provided for each patient. However, corneal biomechanical properties including viscosity, elasticity, and viscoelasticity are much more difficult to be determined *in vivo*. These factors may be changed during the process of certain ocular disorders such as keratoconus and glaucoma,^[1]^ and are claimed to be detectable before their clinical or topographic counterparts in conditions such as forme fruste keratoconus.^[6]^ Therefore, an accurate method to evaluate corneal biomechanics *in vivo* is crucial for predicting corneal surgical outcomes and for optimum surgical planning.^[2]^

Corvis ST corneal parameters may be considered a proxy for actual corneal biomechanical factors; however, because of substantial influences from other determinants such as CCT, keratometry, and particularly IOP,^[4]^ these factors should be interpreted with caution. T1, the time to first applanation, is the factor that has been essentially used for estimating IOP,^[11]^ and hence showed a perfect direct association with IOP [Figure 1]. T1 also showed a weaker direct association with CCT, which reminds the confounding effect of CCT on IOP measurement. Because this factor is closely related to IOP, it is not suitable for use as a proxy for corneal biomechanical properties. V1, T2, T, DA, PD, and R were also more or less affected by IOP. For these parameters (T2 and DA, in particular), an IOP-corrected value (based on the regression analysis) or IOP-stratified charts (such as the one that is shown in Table 6 or those that were provided by Huseynova *et al* ^[1]^) should be used; otherwise, significant mistakes may occur. For example, based on the normative data displayed in Table 2, a DA of 1.24 mm should be considered normal, whereas it is outside normal range for eyes with IOP ≥ 16 mmHg [Table 6].

The present study has provided a reference for normal range of Corvis ST parameters [Tables 2 and 6] in Iranian population. Data from individuals who satisfy the enrolment criteria of this study may cautiously be compared with the provided normal values. In addition to normal range of parameters, we have also provided the normal range of the interocular differences. Because the two fellow eyes are almost symmetric in most topographic and biomechanical properties, an out of range value may prompt further investigations for possible implicit disorders. The interocular ranges are provided as both absolute (95% range of real difference) and relative (absolute variation divided by the mean of the fellow eyes) variations. For relative variation values < 10%, this parameter may be more informative because it incorporates the mean value as well; but for the relative variation > 10% (typically for those with small mean value), the relative variation measurements are exaggerated and useless. Absolute variation values may be more clinically useful for this class of Corvis ST parameters.

Several previous studies have evaluated Corvis ST parameters in normal and abnormal eyes. Hong *et al* reported that Corvis ST demonstrated excellent consistency in IOP measurement perhaps because it might be less affected by corneal properties.^[5]^ Reznicek *et al* reported good repeatability and good accuracy of Corvis ST compared to standardized ultrasound pachymetry or Goldmann applanation tonometry for measuring CCT and IOP in healthy subjects, and in patients with ocular hypertension and glaucoma.^[7]^ The results of our regression analysis of the factors associated with Corvis ST parameters closely parallels the findings of Huseynova *et al*.^[1]^ In both studies, T 1 and R were significantly associated with CCT, and T1, T2, and DA were correlated to IOP.^[1]^ In both studies, T1 and R were significantly associated with CCT. Also, T1, T2, and DA were correlated with IOP.

In a recent study on healthy Brazilian patients, Valbon and colleagues^[11]^ reported a normal range of Corvis ST parameters. Compared to our study, they enrolled fewer patients (*n* = 90), but with broader enrolment criteria (age range: 21 to 79 years). The mean values of Corvis ST parameters that were reported by Valbon *et al* were quite different compared to ours: T1 (8.32 vs 7.36 ms); T (18.38 vs 16.84 ms); T2 (23.80 vs 20.13 ms); L1 (2.07 vs 1.82 mm); L2 (2.37 vs 1.34 mm); DA (1.05 vs 1.05 mm); R (11.09 vs 7.35 mm); V1 (0.21 vs 0.12 m/s); and V2 (–0.33 vs –0.67 m/s), respectively.^[7]^ However, these differences were not unexpected, because their study population enrolled older patients from a different ethnicity. Previous studies have established the role of ethnicity on CCT and IOP,^[12,13]^ the two fundamental determinants of Corvis ST parameters.^[1]^ The differences in Corvis ST values between the two studies further underscores the importance of using customized charts, based on underling ocular and demographic factors, to improve accuracy of detecting abnormal cases in each particular population.

The present study has the advantage of including a large number of cases leading to more precise normative ranges, but it is limited due to its relatively strict enrolment criteria, which reduces the generalizability of the findings. In addition, we did not document the ethnicity. However, our sample was relatively homogenous with the majority of our patients consisting of those with Persian ethnicity. Our results should only be used for the population of refractive surgery candidates with similar age range, ethnicity, and refractive error.

In conclusion, this study has provided a reference normative database for Corvis ST parameters in Iranian refractive surgery candidates, which can be used with caution in selected patients who satisfy the enrolment criteria. Several demographic and ocular factors, and IOP in particular, essentially affected the Corvis ST parameters, and this issue should be considered when interpreting the results.

##  Financial Support and Sponsorship

Nil.

##  Conflicts of Interest

There are no conflicts of interest.
